# Tibio-Talo-Calcaneal Arthrodesis: Evaluation of the Effectiveness of a Specific Surgical Technique in 17 Cases

**DOI:** 10.7759/cureus.62014

**Published:** 2024-06-09

**Authors:** Anass Abaydi, Jihad Radi, Amine Tbatou, Kamal Lahrach, Fawzi Boutayb

**Affiliations:** 1 Orthodontics, University Hospital Center Hassan II, Fès, MAR; 2 Traumatology and Orthopedic Surgery Department A, University Hospital Center Hassan II, Fès, MAR

**Keywords:** hindfoot fusion, tibiotalocalcaneal arthrodesis, corticocancellous bone plugs, retrograde intramedullary femoral nail, ankle arthrodesis

## Abstract

Introduction: Ankle arthrodesis is a crucial surgical intervention for advanced hindfoot conditions, aiming to restore plantigrade walking and alleviate pain. This study evaluates the effectiveness of a specific surgical approach for tibiotalocalcaneal arthrodesis (TTCA), focusing on rigorous risk factor control, corticocancellous grafting, and internal fixation using an angled retrograde femoral nail in the sagittal plane, and assesses the outcomes of this approach in terms of bone fusion and reduction of postoperative complications.

Materials and methods: This retrospective analysis includes 17 patients who underwent TTCA in a trauma-orthopedic department over seven years. Data were collected from medical records, the HOSIX software, and patient consultations. Preoperative assessments, surgical techniques, postoperative care, and follow-up evaluations were documented.

Results: The mean age of patients was 42.4 years, with a male predominance. Surgical indications included post-traumatic arthropathy (53%), inflammatory arthropathy, ankle infectious pathologies, and Charcot foot and ankle prosthesis failures. All patients underwent standard preoperative evaluations and received corticocancellous grafts. An angled retrograde femoral nail in the sagittal plane was used for internal fixation. Postoperative immobilization lasted 6 to 8 weeks, with subsequent rehabilitation. The bone fusion rate was 100%, with a low complication rate (23.5%).

Discussion: Our study showed a younger patient population with a male predominance, different from some previous studies. Surgical techniques, including the anterior approach combined with a lateral subtalar approach, were consistent with some studies but differed from others. Corticocancellous grafts and the angled retrograde femoral nail in the sagittal plane demonstrated favorable outcomes in terms of fusion. Complication rates were lower compared to some previous reports, highlighting potential improvements in postoperative management.

Conclusion: The surgical approach described for TTCA, emphasizing rigorous risk factor control, corticocancellous grafting, and internal fixation using an angled retrograde femoral nail in the sagittal plane, led to satisfactory bone fusion and reduced postoperative complications. These results underscore the importance of this approach in achieving optimal functional outcomes in ankle arthrodesis.

## Introduction

Ankle arthrodesis is a surgical procedure aimed at achieving a stable fusion of the joint. It is primarily indicated in cases of advanced rearfoot joint damage and structural abnormalities [1]. This procedure is considered a last-resort option after exhausting conservative treatments, to restore pain-free joints, adequate stability, and a normal gait with plantigrade support. The success of this procedure is closely linked to the precise application of a technique that ensures anatomically correct fusion of the ankle in all three planes of space [1,2].

In the modern landscape of orthopedic surgery, ankle arthrodesis remains a crucial option compared to ankle replacement for treating advanced conditions of this joint. Despite a potential risk of overloading adjacent joints, which may lead to secondary osteoarthritis, this method offers satisfactory functional advantages and a reduced risk of complications, making it a preferred choice in many cases [2].

Among the various surgical approaches, tibio-talocalcaneal arthrodesis using a retrograde nail holds a prominent place in the therapeutic arsenal. This procedure aims to achieve fusion of the tibiotalar and talocalcaneal joints using a locked retrograde nail known for its reliability, particularly in complex situations such as bipolar ankle arthrodesis [1].

The primary objectives are to describe the effectiveness of a specific surgical approach for tibio-talocalcaneal arthrodesis (TTCA). This approach focuses on the rigorous control of risk factors, the use of corticospongious grafts, internal fixation with an angled retrograde femoral nail in the sagittal plane, and evaluating the outcomes of this approach in terms of bone fusion and reduction of postoperative complications.

## Materials and methods

This study is a retrospective analysis involving 17 patients who underwent tibio-talocalcaneal arthrodesis in the Trauma-Orthopedics Department A at CHU Hassan II in Fez over seven years, from January 1, 2017, to December 30, 2023. The average follow-up duration was 35.5 months (ranging from 11 to 60 months), with a minimum follow-up of 11 months.

Data were carefully gathered from a variety of sources, including hospitalization records, medical files, the HOSIX program, and patient consultations. To guarantee complete data acquisition, a structured data collection sheet was created, which was designed to record key study parameters. These parameters encompassed epidemiological data such as patient age, gender, and comorbidities, clinical examination data including pain levels, range of motion, and functional status, and radiological data detailing pre- and postoperative imaging findings.

**Figure 1:**
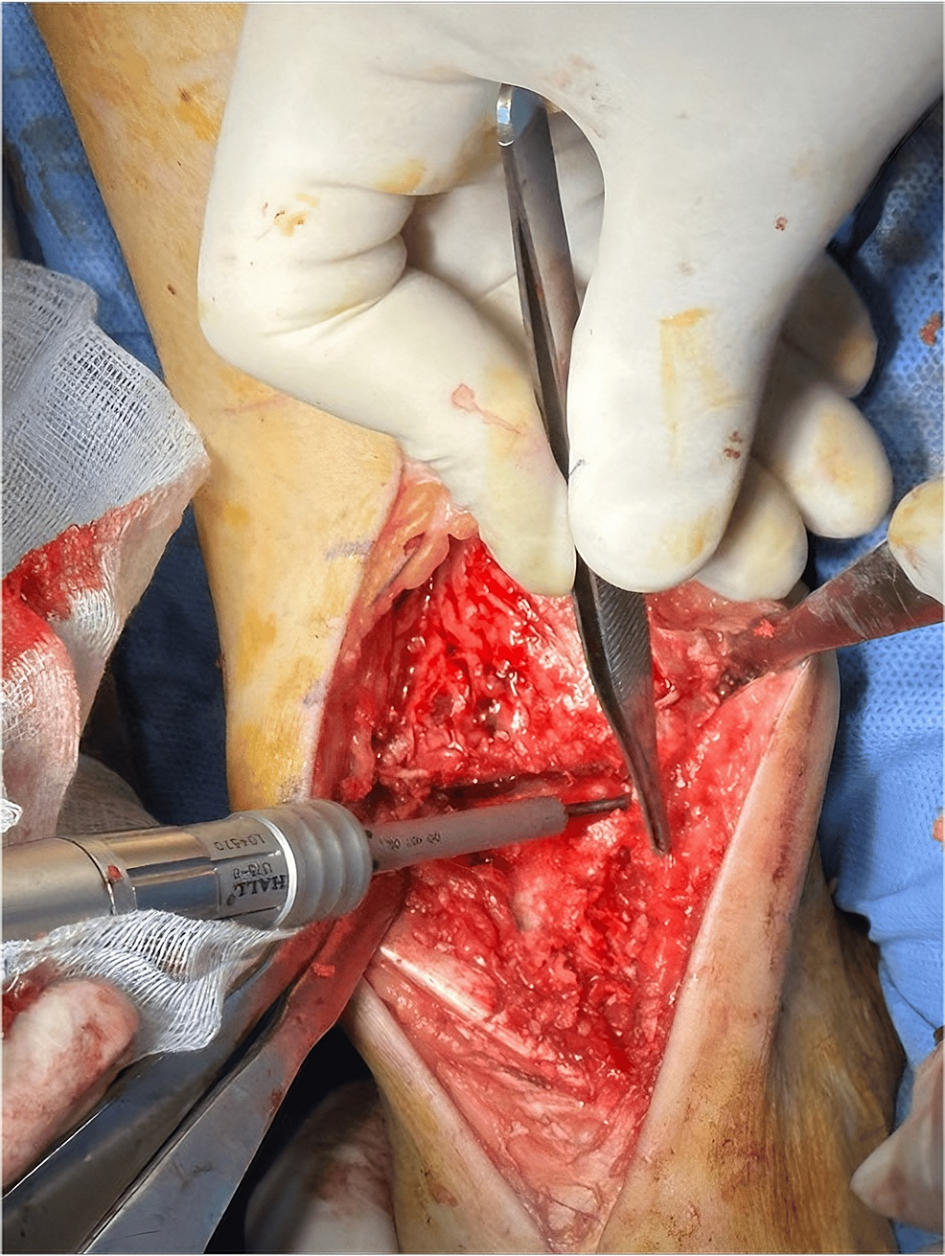
Conventional anterior approach with opening of the joint and debridement of the articular surfaces.

**Figure 2:**
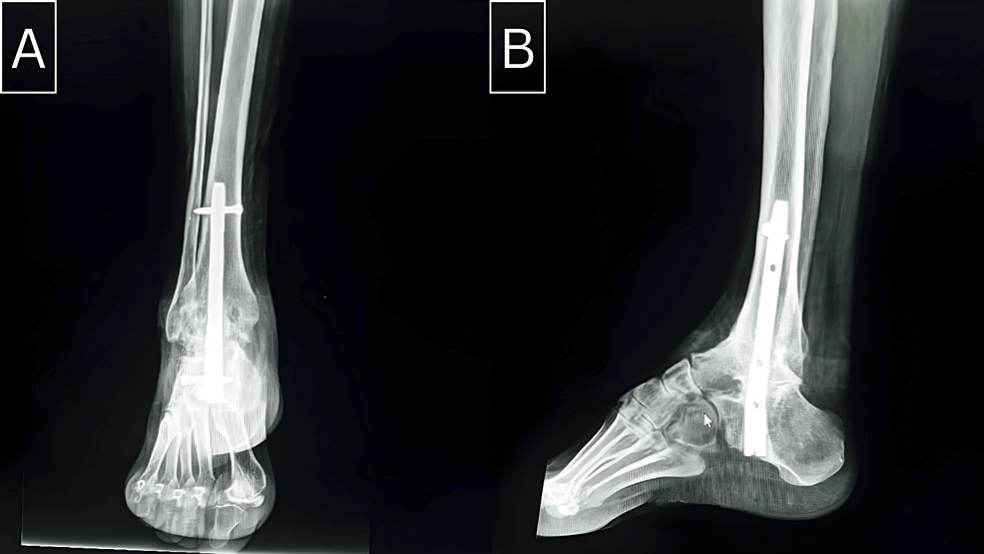
Weight-bearing anteroposterior (A) and lateral (B) X-ray of the ankle five months after tibio-talo-calcaneal arthrodesis with a retrograde femoral nail.

The indication for tibio-talocalcaneal arthrodesis was established based on the presence of painful tibio-talocalcaneal osteoarthritis. This diagnosis was confirmed through anteroposterior and lateral ankle radiographs, which provided crucial visual evidence of joint degeneration. All patients underwent surgery via a classical anterior approach to the ankle (Figure 1), a technique that allows optimal access for the procedure. The surgical method involved transfixing retrograde nail osteosynthesis, a robust approach for achieving joint stability and was consistently accompanied by bone grafting to promote osteointegration and enhance the likelihood of successful fusion (Figure 2).

Rehabilitation began immediately after cast removal, focusing primarily on the forefoot joints and restoring a plantigrade gait.

The postoperative evaluation was comprehensive and included both clinical and radiological assessments. Clinically, the evaluation criteria focused on functional outcomes such as walking distance, ankle stability during walking, and the presence or absence of pain, which are pivotal for assessing the success of the arthrodesis in improving patient quality of life. Radiologically, anteroposterior and lateral radiographs were utilized to monitor the consolidation and alignment of the ankle joint (Figure 2). Additionally, computed tomography (CT) scans were employed selectively to confirm joint integrity and assess for any subtle complications not evident on standard radiographs, although CT was not used systematically for all patients.

All complications, including later complications like non-union or malunion, as well as acute postoperative problems like infection, were carefully recorded. Based on a mix of clinical complaints and paraclinical evidence, each patient's progression was monitored, offering a comprehensive picture of the results of tibio-talocalcaneal arthrodesis in this patient group.

Subjective evaluation was conducted by assessing patient satisfaction, which was categorized as satisfied, moderately satisfied, or not satisfied. The objective evaluation utilized the American Orthopedic Foot and Ankle Score (AOFAS).

## Results

During the defined period, 17 patients underwent surgery. The average age at the time of surgery was 42.4 years (ranging from 21 to 71 years), with a male predominance, as indicated by a sex ratio of 0.7 (F/M). The surgery involved the right ankle in 70.5% of cases.

Among the medical histories, five patients were diabetic, three of whom were poorly controlled, seven were smokers, one patient had end-stage chronic renal failure, and another was obese. 65% of our patients had a history of at least one previous ankle surgery.

**Figure 3:**
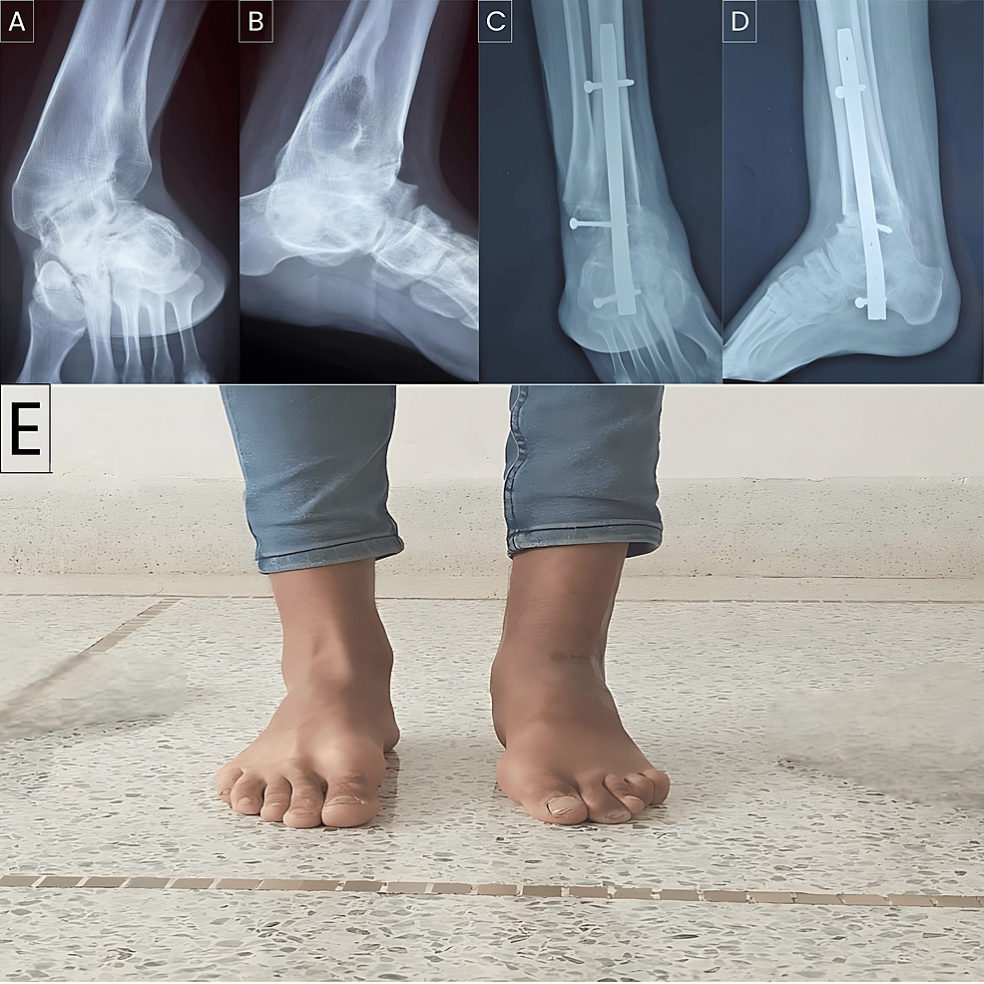
Charcot's disease is complicated by the sequelae of septic arthritis. Anteroposterior (A) and lateral (B) X-ray images of the ankle show malunion of the ankle and a geode in the distal quarter of the tibia. Anteroposterior (C) and lateral (D) X-ray images of Tibio-talo-calcaneal arthrodesis with retrograde femoral nail: results nine months postoperatively. E: Functional outcome nine months after tibio-talo-calcaneal arthrodesis

**Figure 4:**
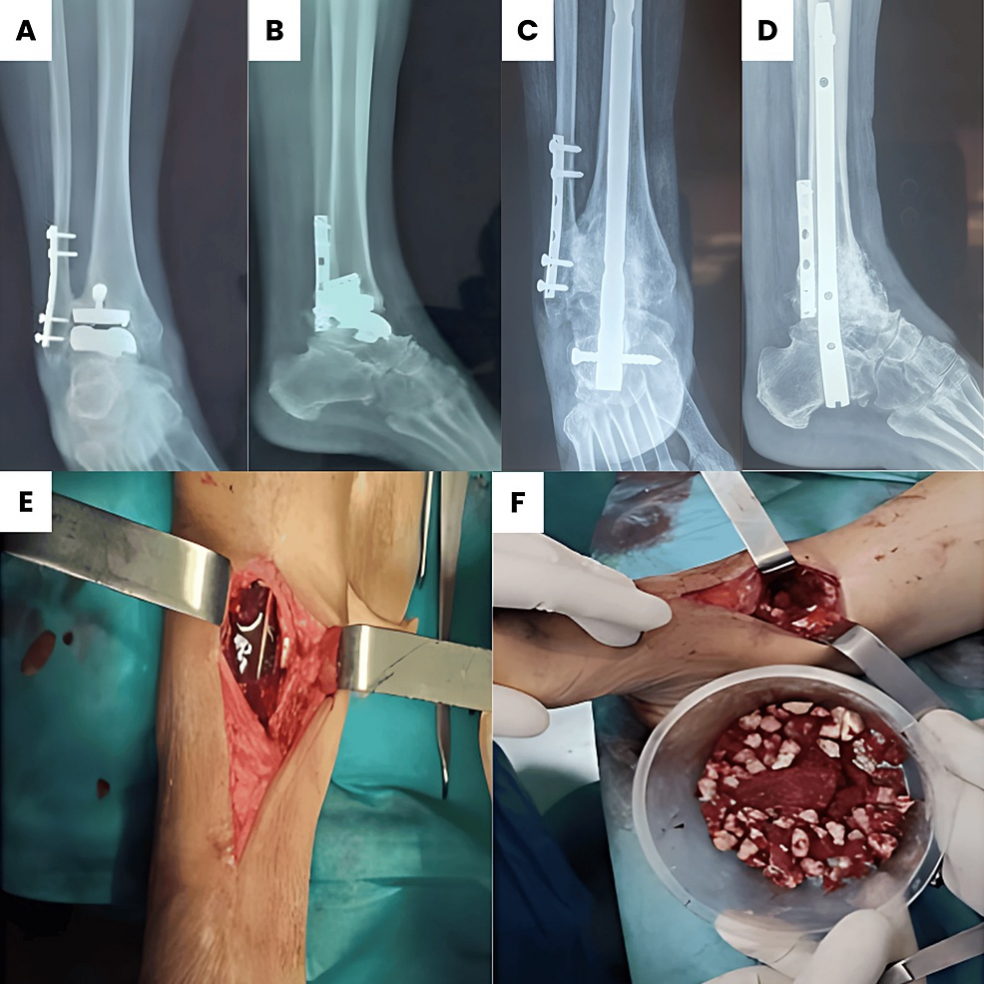
Management of loose total hip prostheses. Antero-posterior (A) and lateral (B) X-rays of the ankle show a loose TKA. Autograft and allograft to fill massive bone loss after TKA removal (E, F). Antero-posterior (C) and lateral (D) X-rays of the ankle 16 months after TTCA with retrograde femoral nail.

The etiologies of tibio-talocalcaneal arthrosis were predominantly post-traumatic arthropathy, representing 53% of cases. This included five talus fractures, one talus enucleation, four bipolar post-traumatic arthropathies (three tibial pilon fractures + calcaneal fracture, and one trimalleolar fracture + calcaneal fracture), one inflammatory arthropathy, two infectious ankle pathologies, two Charcot feet (Figures 3A, 3B), and two cases of loose ankle prostheses (Figures 4A, 4B).

All patients underwent standard anteroposterior and lateral ankle radiographs, with CT scans performed in 76% of them. Angiography of the lower limbs was conducted in 53% of patients with preexisting arteriopathy risk or lower limb vascular axis involvement, notably in diabetics or in cases of open fractures or high-energy trauma.

Preventive measures were taken before surgery for at-risk patients, including better glycemic control for diabetics, cessation of smoking for at least one month before surgery, and ensuring no infections for at least two weeks before surgery.

**Figure 5:**
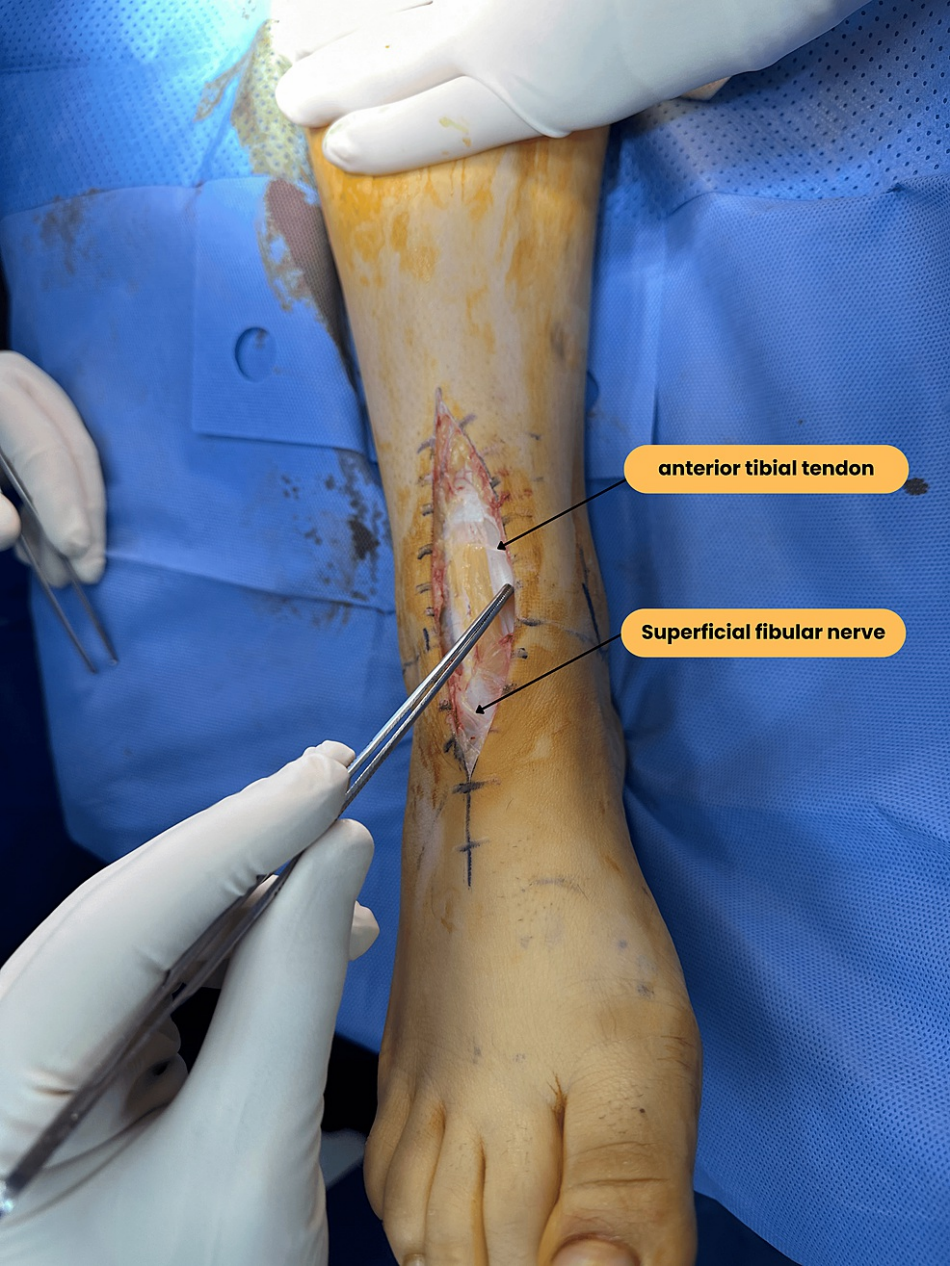
Anterolateral approach to the ankle

All surgeries were performed by the same surgeon. The most common surgical approach was the classical anterior approach (Figures 1, 4C, 4D) with a lateral subtalar approach, performed in 15 patients (88%), while two patients underwent an antero-external approach (Figure 5) and a medial approach for corrective osteotomies.

All patients received grafts in the tibiotalar and subtalar joints using corticospongious autografts harvested from the iliac crest. Two patients required additional allogenic grafts to fill significant bone loss (Figures 4E, 4F).

Three patients required associated procedures: two required proximal tibiofibular closing wedge osteotomies, and one required talar head resection and talonavicular arthrodesis as part of tibiotalocalcaneal arthrodesis due to flatfoot with medial instability.

**Figure 6:**
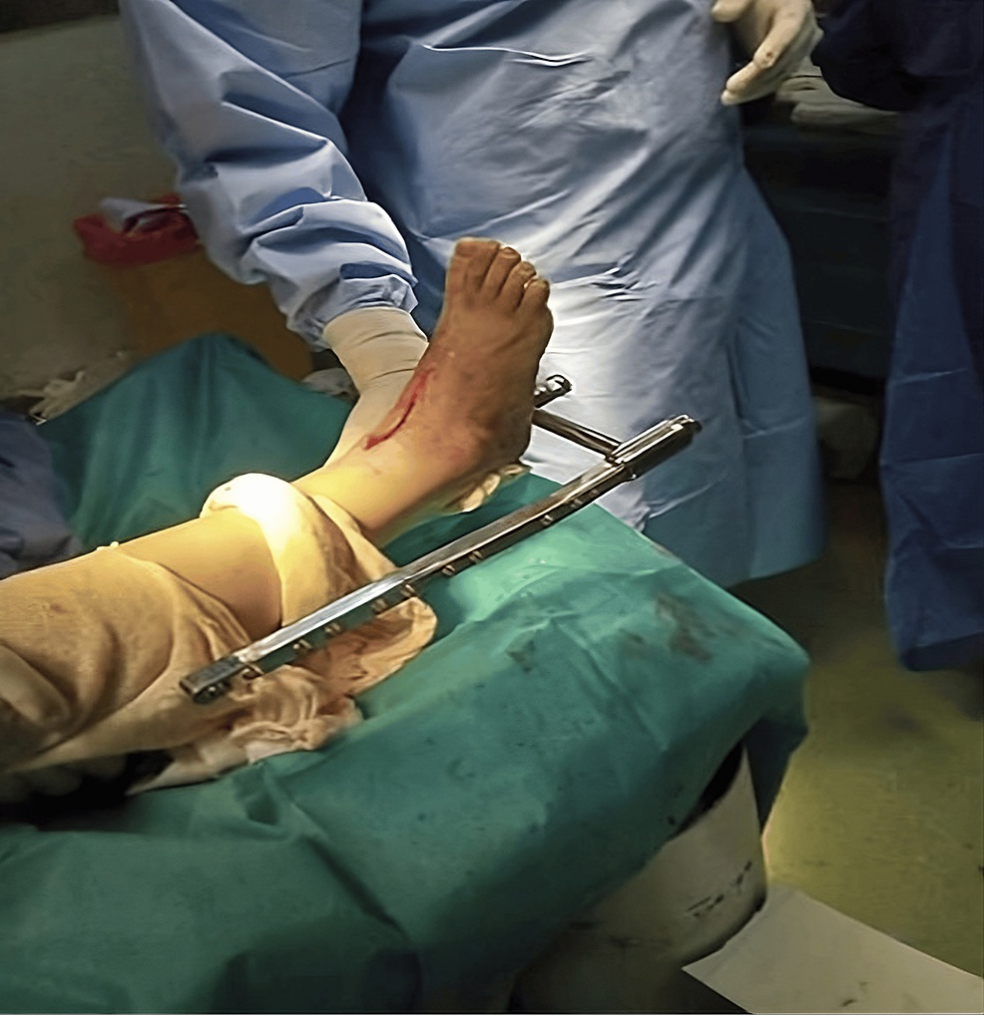
Intraoperative image of retrograde femoral nail application

The retrograde nail used in our study was a retrograde femoral nail due to the unavailability of a dedicated retrograde ankle nail. It was an anteroposterior beaked nail with lateral locking (Figures 2, 3C, 3D, 4C, 4D, 6).

All patients were immobilized with a posterior plaster splint for 6 to 8 weeks. Weight-bearing was prohibited for 8 to 12 weeks, then partial weight-bearing was allowed for two weeks before permitting full weight-bearing. Rehabilitation focused on restoring a stable and plantigrade gait.

The bone-fusion rate was 100%. We noted two superficial infections in one diabetic patient and one patient with chronic renal failure, necessitating rehospitalization for antibiotic therapy and dressing control. One patient experienced cutaneous suffering and delayed healing. A delay in consolidation was observed in one smoker. No cases of pseudarthrosis or hardware failure were noted at the last follow-up.

The overall complication rate was 23.5%, with no high-grade complications. Six patients had limb length discrepancies, including one significant case with a 5 cm discrepancy.

94% of our patients were satisfied or very satisfied, except for a 24-year-old female patient with a significant postoperative limb length discrepancy of 4 cm. The AOFAS score improved from 25 ± 13 to 72.2 ± 13 (Figure 3E).

## Discussion

Based on our findings, the average age of the patients was 42.4 years, which differs from the demographic characteristics observed in other studies. Our study shows a younger population and a higher male prevalence, while the studies by Laffenêtre et al. [1] and McLeod Phillips et al. [2] include an older population with a more balanced gender distribution. The study by Bing Howe Lee et al. [3] presents similar characteristics to ours in terms of average patient age, but with a slight female prevalence.

65% of our patients had a history of previous ankle surgery. This observation is consistent with the results of Laffenêtre et al. [1] as well as a meta-analysis conducted by Jehan et al. [4], which explains the complexity of cases undergoing this type of treatment.

Post-traumatic arthropathy is the most common cause in the literature, which is consistent with our results. Primary osteoarthritis represents the second cause of TTCA, but it is the least frequent etiology in our study.

The classical anterior approach combined with a lateral subtalar approach was used by Laffenêtre et al. [1] as well as in our study. This contrasts with the majority of articles in the literature, particularly those from American and Asian sources [2,3,5], which favor the lateral approach centered on the fibula.

Wenbao He et al. [5] compared the results of three groups and concluded that fibular grafting can shorten fusion time compared to fibular osteotomy and fibular preservation. Boer et al. [6] concluded that formal debridement of the subtalar joint is not necessary for successful fusion, which contradicts the findings of Gross et al. [7], who reported a fusion rate of 86% at the talo-tibial joint and 74% at the subtalar joint without debridement.

All our patients underwent tibiotalar and subtalar debridement with preservation of the fibula, except for two patients who underwent fibular osteotomy as part of the correction of hindfoot deformities. [8]

Arthroscopic arthrodesis was first described in 1983 [9] and has since become a common technique due to its significantly less invasive nature and minimal periarticular tissue damage [10]. In 2010, Devos Bevernage et al. introduced a new minimally invasive posterior arthroscopic TTCA procedure [11]. According to L. Lingnau et al., arthroscopic arthrodesis is a safe and reproducible procedure without increased operative complications [12]. It plays a role in TTCA, especially in cases of poor skin conditions and multiple previous ankle surgeries. However, it is not suitable for cases of significant hindfoot displacement or deformity [11].

Due to our lack of experience in ankle arthroscopy, none of our patients underwent arthroscopic arthrodesis.

Bone grafts are commonly used in foot and ankle reconstructive surgeries [13]. They help promote fusion [14,15]. Currently, the gold standard procedure is the corticocancellous autograft, which has osteoconductive, osteoinductive, and osteogenic properties [16,17]. M. Tricot et al. concluded that using a combined autograft-demineralized bone matrix or allograft-demineralized bone matrix-marrow aspiration results in comparable outcomes in both cases [18].

In our study, all patients received autografts harvested from the iliac crest. Two patients also received autografts from the tibial and fibular closure wedge osteotomies for deformity correction. In addition, two patients with significant bone loss during total ankle prosthesis loosening required filling with allografts from the femoral head bone bank combined with iliac crest autografts. Laffenêtre et al. added BMP to the allograft to reliably consolidate the allograft-host bone junction [1].

Various retrograde nails are used for the TTCA. They are classified into two classes: non-angled retrograde nails and retrograde nails angled in the coronal plane, dynamic retrograde nails, and static retrograde nails.

Laffenêtre did not find a significant difference in the fusion rate achieved in his series compared to a meta-analysis (86.7% over 641 cases) within the same timeframe and in the series of angled nails by Budnar et al. (89%) [1,4,8].

Several studies have aimed to emphasize the advantages of using angled nails to prevent injury to the lateral plantar pedicle and to achieve a higher rate of bone fusion [19-21].

Muckley et al. found no significant difference between angled and non-angled nails in terms of plantar injuries. They concluded that a wide plantar incision and the use of soft tissue protection can reduce the risk of complications, regardless of the type of nail used [22].

In a study by Michel et al., it was found that the consolidation time and fusion rate of the ankle joint were higher in the dynamic group, but this was not statistically significant [23].

In our study, due to the unavailability of a dedicated retrograde ankle nail, we used a retrograde femoral nail angled at 10° in the sagittal plane. We observed no plantar neurovascular injuries and achieved a 100% fusion rate.

Our study revealed a lower overall complication rate compared to previous research. Only 23.5% of our patients experienced minor complications that did not require surgical intervention. In contrast, Laffenêtre et al. reported an overall complication rate of 65%, with 22% high-grade complications and a 16% reoperation rate [1]. McLeod et al. initially suggested an increased complication rate of 50.5% [2]. Ramelt et al. found a complication rate of 23.7% [24], while a smaller study by Pinzur and Noonan noted a complication incidence of 22% [25]. Our results indicate a potential improvement in the management of postoperative complications.

Recent literature by Myers et al. has shown that tight and long-term glycemic control leads to improved outcomes and fewer surgical site infections in diabetic patients [26]. However, McLeod et al. found no difference between diabetic and non-diabetic patients. Pearson RG et al. suggest that quitting smoking before surgery benefits fracture healing [27]. Currently, complete smoking cessation for three months before any TTCA is recommended, not only in complex reconstruction situations [1,28].

Infection control is essential for preventing the spread of infection through the nail with osteomyelitis, whether it is of infectious origin or aseptic [29].

Smokers were required to quit smoking at least one month before surgery; otherwise, the operation could not be performed. Diabetics also had to ensure meticulous glycemic monitoring in collaboration with an endocrinologist for optimal glycemic control. Biological tests were conducted to exclude any infection, particularly in patients with Charcot disease or sequelae of infectious arthritis of the ankle. No signs of infection were detected for at least two weeks before the procedure.

Francesco Franceschi et al. found that hardware removal was necessary for 77 out of 862 tibio-talo-calcaneal arthrodeses [30]. In our study, no hardware removal was performed up to the last follow-up.

The AOFAS score improved from an average of 25 to 72.2, with 94% of our patients reporting satisfaction, aligning with existing literature.

Our study was limited in the number of included patients. Additionally, we excluded patients with comorbidities, which could result in a selection bias for surgery.

## Conclusions

Ankle arthrodesis is a critical surgical procedure for advanced hindfoot conditions, to restore joint function and relieve pain. Our study has shown that a meticulous surgical approach involving strict management of risk factors, the use of corticocancellous bone grafts, and internal fixation with a retrograde femoral nail angled in the sagittal plane results in satisfactory bone fusion and reduced postoperative complications. These findings underscore the importance of this methodology in achieving optimal functional outcomes in ankle arthrodesis.

Our method led to a 100% bone healing rate, with a low rate of complications compared to other studies. The high level of patient satisfaction, as indicated by the AOFAS score improving from 25 ± 13 to 72.2 ± 13, and the low occurrence of minor complications highlight the effectiveness of this technique. These findings support recent literature that highlights the advantages of bone grafting techniques and retrograde nail fixation in improving fusion rates and reducing postoperative complications. Ankle arthrodesis performed using this combined approach is a robust and effective solution for severe hindfoot conditions, significantly enhancing patient functionality and quality of life.
